# The Long Non-Coding BC200 Is a Novel Circulating Biomarker of Parathyroid Carcinoma

**DOI:** 10.3389/fendo.2022.869006

**Published:** 2022-05-02

**Authors:** Annamaria Morotti, Filomena Cetani, Giulia Passoni, Simona Borsari, Elena Pardi, Vito Guarnieri, Chiara Verdelli, Giulia Stefania Tavanti, Luca Valenti, Cristiana Bianco, Stefano Ferrero, Sabrina Corbetta, Valentina Vaira

**Affiliations:** ^1^Department of Pathophysiology and Transplantation, University of Milan, Milan, Italy; ^2^Division of Pathology, Fondazione IRCCS Ca’ Granda-Ospedale Maggiore Policlinico, Milan, Italy; ^3^Endocrine Unit 2, University Hospital of Pisa, Pisa, Italy; ^4^Department of Clinical and Experimental Medicine, University of Pisa, Pisa, Italy; ^5^Division of Medical Genetics, Fondazione IRCCS Casa Sollievo della Sofferenza, Foggia, Italy; ^6^Laboratory of Experimental Endocrinology, IRCCS Istituto Ortopedico Galeazzi, Milan, Italy; ^7^Endocrinology and Diabetology Service, IRCCS Istituto Ortopedico Galeazzi, Milan, Italy; ^8^Department of Biomedical, Surgical and Dental Sciences, University of Milan, Milan, Italy; ^9^Precision Medicine – Department of Transfusion Medicine and Hematology, Fondazione IRCCS Ca’ Granda Ospedale Maggiore Policlinico, Milan, Italy

**Keywords:** parathyroid, epigenetics, long non-coding RNAs, brain cytoplasmic RNA 1, biomarker, parathyroid carcinoma

## Abstract

Long non-coding RNAs (lncRNAs) are an important class of epigenetic regulators involved in both physiological processes and cancer development. Preliminary evidence suggested that lncRNAs could act as accurate prognostic and diagnostic biomarkers. Parathyroid cancer is a rare endocrine neoplasia, whose management represents a clinical challenge due to the lack of accurate molecular biomarkers. Our previous findings showed that human parathyroid tumors are characterized by a different lncRNAs signature, suggesting heterogeneity through the different histotypes. Particularly, we found that the lncRNA BC200/BCYRN1 could represent a candidate biomarker for parathyroid carcinomas (PCas). Here we aimed to extend our preliminary data evaluating whether BC200 could be an accurate non-invasive biomarker of PCas to support the clinical management of patients affected by parathyroid tumors at diagnosis, prognosis and follow-up. To provide a non-invasive point-of-care for parathyroid carcinoma diagnosis and follow-up, we analyzed BC200 expression in patients’ serum through digital PCR. Our results show that BC200 counts are higher in serum from patients harboring PCa (n=4) compared to patients with parathyroid adenoma (PAd; n=27). Further, in PAd patients circulating BC200 levels are positively correlated with serum total calcium. Then, we found that BC200 is overexpressed in metastatic PCas (n=4) compared to non-metastatic ones (n=9). Finally, the lncRNA expression in PCa patients’ serum drops are reduced after parathyroidectomy, suggesting its possible use in the post-operative setting for patients follow-up. Overall, these findings extend the knowledge on BC200 in parathyroid tumors, supporting its role as a useful biomarker for management of PCa.

## Introduction

Parathyroid carcinoma (PCa) is a rare endocrine disease representing <1% of the parathyroid gland’s tumors, which may led to elevated parathyroid hormone (PTH) secretion, hypercalcemic crisis, bone disorders, renal failure and a consequent fatal hyperparathyroidism (1). Opposite to other endocrine tumors, it occurs with an equal frequency in men and women (2). Further, in about half of the cases the current clinical approach, i.e. surgery followed by adjuvant therapies, is not curative and tumor relapse often occurs. This results in a poor survival rate, with 85% of the patients dying within 5 years (3). Moreover, the presence of a metastatic lesion at diagnosis and the tumor size are worse prognostic factor (4, 5).

Current diagnosis of parathyroid carcinoma is made intra- and post-operatively and it is based on advanced histopathologic criteria of an invasive phenotype, like vascular invasion or distant metastasis (6). Histologically, the absence of the Cell Division Cycle 73 (CDC73), amplification of the Cyclin D1 (CCND1) gene and CCND1 overexpression, are the most common molecular features distinguishing PCas from benign parathyroid adenomas (PAds). In addition, the mutational status of APC, RB, E-cadherin, MDM2 and p53 overexpression are considered useful biomarkers for parathyroid carcinomas’ diagnosis (7). Nevertheless, some PCas have an indolent growth and mimic benign lesions making their diagnosis more challenging (8). This clinical scenario is further complicated by the absence of accurate molecular markers supporting the differential diagnosis between parathyroid adenomas and carcinomas. We previously showed that non-coding RNAs play important roles in parathyroid tumorigenesis, acting both as oncogenes (9) and as biomarkers (10, 11).

In particular, long non-coding RNAs (lncRNAs) actively participate in endocrine tumors by promoting cancer cell proliferation, migration and metastasis (12, 13). Moreover, lncRNAs are involved in drug resistance and correlate with patient’s prognosis and overall survival (14); hence the evidence of lncRNAs as prognostic and diagnostic biomarkers. In recent years, lncRNAs became a crucial topic in parathyroid tumors field, with the purpose of identifying biomarkers able to distinguish malignant from benign glands (11, 15–17). Recently, we profiled 90 lncRNAs in a cohort of 24 PAds, 9 PCas and 4 normal glands, and we identified Brain Cytoplasmic RNA 1 BC200 (BCYRN1) as the most upregulated lncRNA in PCas compared to the other histotypes (11). BC200 is the shortest and neuronal-specific lncRNA whose aberrant expression was originally associated to neurodegenerative disorders, but its deregulation has been described also in cancer (18). Particularly, its expression is dramatically increased in gastric, colorectal and breast cancers, where it positively correlates with tumor size and clinical stage. In these contexts, BC200 affects cancer cells motility, promoting *in vitro* cell migration and proliferation (19–21). Interestingly, BC200 was detected in the circulation of patients with invasive breast cancer compared to healthy donors, suggesting its possible role as a non-invasive tumor biomarker (22).

The identification of biomarkers, especially non-invasive ones, in parathyroid tumors is key for their correct diagnosis, prognosis and patients’ follow-up. Based on our preliminary evidence, here we aimed to get further insights on the possible use of BC200 as a biomarker of parathyroid tumor malignancy.

## Materials and Methods

### Ethical Approval and Consent to Participate

This research was performed in accordance with the World Medical Association Declaration of Helsinki. The study was approved by the Ethical Committee of Scientific Institute San Raffaele Hospital (#CE40/2019) and written informed consent was obtained from all the participants.

### Parathyroid Samples

Thirteen PCa tissues were collected and analyzed for BC200 mRNA expression. Histological diagnosis of PCas was established according to WHO guidelines and as previously described (11, 23). Fasting serum total and ionized calcium were measured by a multichannel autoanalyzer. Intact circulating PTH was determined by a chemiluminescent immunoassay (Nichols Advantage, Nichols Institute Diagnostics, San Clemente, CA, USA) in order to diagnose PHPT. Serum 25-hydroxyvitamin D (250HD) was assayed by Diasorin.

Blood serum was collected from PAds (n=27) and PCas (n=4, three of which matched pre- and post-surgery), following the standard procedures. In brief, whole blood was harvested in a 5 ml tube and immediately centrifuged at 1000 rcf for 15 minutes at +4°C. Serum was then transferred into 1.5 ml Biopur (Eppendorf) and stored at -80°C until use. PAds and PCas clinical data were reported in **Tables 1**, **2**, respectively.

**Table 1:** Clinical and biochemical data of parathyroid adenomas.

Patient ID	Sex	Age at diagnosis	250HD (ng/ml)	PTH (pg/ml)	Ca^2+^ (mmol/L)	Total calcium (mg/dl)	Creatinine (mg/dl)
PAd1	M	73	13,7	413	1.91	12.9	1.18
PAd2	F	61	29,4	NA	1.38	10.2	NA
PAd3	M	77	NA	140	NA	11.8	1.64
PAd4	F	72	17,8	167	NA	11.3	NA
PAd5	F	68	6,9	213	NA	10.7	0.74
PAd6	F	49	35,4	203	1.08	9.2	1.01
PAd7	F	83	12,0	740	NA	13.3	1.10
PAd8	F	61	17,9	78	1.36	10.3	0.55
PAd9	F	69	15,0	261	1.47	11.9	0.68
PAd10	F	61	25,0	142	1.40	10.0	0.56
PAd11	M	60	15,0	183	1.53	11.1	1.09
PAd12	F	60	14,3	180	1.13	8.6	0.79
PAd13	F	72	26,0	180	1.40	10.9	0.69
PAd14	M	48	23,4	87	1.45	10.8	1.24
PAd15	F	70	32,0	304	1.48	11.4	0.83
PAd16	F	55	20,1	130	1.46	10.7	0.89
PAd17	F	70	19,0	106	NA	11.2	0.70
PAd18	F	50	13,8	149	1.42	10.7	0.55
PAd19	M	52	15,6	103	1.47	11.7	0.83
PAd20	M	48	4,6	544	1.63	11.6	0.55
PAd21	F	54	4,0	401	1.39	10.5	0.84
PAd22	F	76	5,5	66	1.39	10.8	0.85
PAd23	M	66	30,6	142	1.26	11.0	1.19
PAd24	F	58	28,8	41	1.05	9.5	0.64
PAd25	F	46	NA	104	1.49	11.7	0.60
PAd26	M	64	6,4	149	1.39	11.5	0.77
PAd27	F	78	21,8	176	NA	11.4	NA

F, Female; M, Male; NA, Not Available; 250HD, serum 25-hydroxyvitamin D levels; PTH, circulating parathormone levels; Ca^2+^, ionized calcium levels; Total calcium, serum total calcium levels.

Normal values: 250HD 20 and 40 ng/mL; serum PTH 10 to 65 pg/ml; ionized calcium 1.13 to 1.29 mmol/L; serum total calcium 8.4 to 10.4 mg/dl; creatinine 0.74 to 1.5 mg/dL.

**Table 2:** Clinical and biochemical data of parathyroid carcinomas.

Patient ID	Sex	Age at diagnosis	250HD (ng/ml)	PTH (pg/ml)	Ca^2+^ (mmol/L)	Total calcium (mg/dl)	Creatinine (mg/dl)
**Serum**							
PCa1	M	59	NA	>1000	1.99	NA	NA
PCa2	M	49	20,5	2058	1.94	14	NA
PCa3	M	53	23	424	2.1	16.4	0.92
PCa4	M	42	NA	1654	NA	23	NA
**Tissues**							
PCa2	M	49	20,5	2058	1.94	14	NA
PCa5	M	53	NA	63	1.58	11.2	0.58
PCa6	F	60	NA	96	1.56	12.2	1.06
PCa7	M	49	20,5	167	1.54	11.4	NA
PCa8	F	58	9,2	672	1.8	12.7	0.58
PCa9	F	NA	NA	NA	NA	NA	1.06
PCa10	M	38	NA	154	NA	12.9	NA
PCa11	M	57	12	690	2.13	13.9	2.43
PCa12	M	61	NA	391.7	2.92	NA	NA
PCa13	F	31	41	77	1.34	9.1	0.5
PCa14	M	53	NA	444	NA	12.5	NA
PCa15	F	60	NA	275	2	11.7	0.77
PCa16	F	49	NA	293	2.1	16.1	0.9

F, Female; M, Male; NA, Not Available; 25OHD, serum 25-hydroxyvitamin D levels; PTH, circulating parathormone levels; Ca^2+^, ionized calcium levels; Total calcium, serum total calcium levels.

Normal values: 250HD 20 and 40 ng/mL; serum PTH 10 to 65 pg/ml; ionized calcium 1.13 to 1.29 mmol/L; serum total calcium 8.4 to 10.4 mg/dl; creatinine 0.74 to 1.5 mg/dL.

### RNA Purification

Frozen tissues were mechanically dissociated (TissueLyser; Qiagen), and total RNA was purified using Trizol reagent (Invitrogen, Thermo Fisher Scientific, Waltham, MA, USA). Genomic DNA contamination was removed by DNase I Amplification Grade treatment (Invitrogen, Thermo Fisher Scientific).

Total RNA was isolated from 200 µl patient’s serum using miRNeasy Plasma/Serum Advanced kit (217204; Qiagen), according to the manufacturer’s instruction.

### Quantitative RT-PCR (qRT-PCR) Analysis

300 ng of total RNA were reverse transcribed using SuperScript IV Reverse Transcriptase and the High-Capacity cDNA Reverse Transcription Kit (both from Thermo Fisher Scientific). The sequence of the BCYRN1/BC200 TaqMan gene expression assay was previously published (11). B2M was used as internal control for BC200 relative quantification using the comparative Ct method. Then, raw data were median-normalized and log2 transformed for statistical analyses.

### Digital PCR (dPCR)

For the digital PCR assay, 2 ng of the total RNA were reverse transcribed using SuperScript IV Reverse Transcriptase and the High-Capacity cDNA Reverse Transcription Kit, as above. For each sample, 14 µl PCR mix were loaded on the QuantStudio™ 3D Digital PCR 20K Chip Kit v2 (A26316; Applied Biosystem, part of Thermo Fisher Scientific) using the QuantStudio 3D digital PCR Chip Loader. PCR mix was composed as follow: 8 µl of QuantStudio™ 3D Digital PCR Master Mix v2 (A26358; AppliedBiosystem), 0.8 µl of BC200 TaqMan probe, 2.5 µl of cDNA and 3.9 µl of DNA/RNAse-free water/sample. cDNA was amplified using the ProFlex™ 2× Flat PCR system and the reaction underwent the following thermal cycles: 96°C for 10 min; 56°C for 2 min and 98°C for 30 sec (x45 cycles); 60°C for 2 min; 10°C endless. Droplets were read with the FAM channel. No-template control was included in every run.

### Statistical Analysis

Statistical analysis was performed using GraphPad Prism version 7.0 for Windows (GraphPad Software, La Jolla, California, USA). Digital PCR data were analyzed with QuantStudio™ 3D Analysis Suite™ Cloud Software (V3.1) The default confidence level (%) is 95% and the default desired precision (%) is 10%. To determine differences in gene expression, the non-parametric two-tailed Mann-Whitney U-test was performed. To evaluate the power and accuracy of BC200 in discriminating between PAds and PCas, we calculated the Area Under Curve (AUC), using the Receiver-Operating Characteristic (ROC) curve. An AUC >0.7 was considered acceptable. For the correlation analysis, the Pearson r was determined. A p<0.05 was considered statistically significant.

## Results

### BC200 Is Overexpressed in the Serum of PCas Compared With PAds

**Figure 1:**
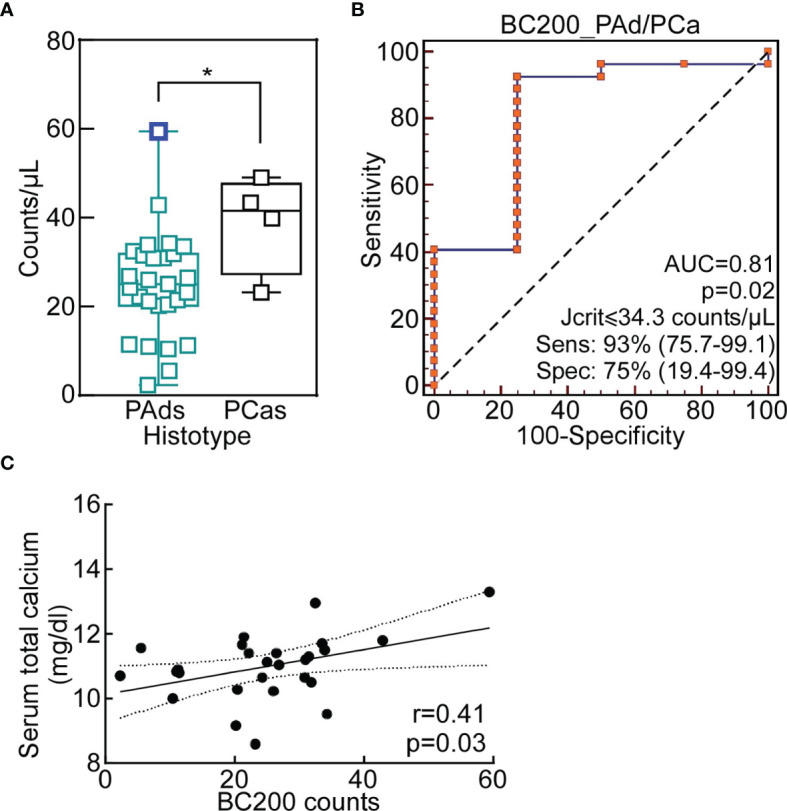
Circulating BC200 expression discriminates between parathyroid adenomas and carcinomas. **(A)** BC200 expression levels in PAds and PCas serum. Each sample is a square and data are presented as box-plot with median. Whiskers represent min to max values. *, p=0.04 from Mann-Whitney U-test. The blue square indicates the PAd7. **(B)** The ROC analysis with the Youden criterion was used to identify the optimal cut-off to differentiate PAds from PCas. P=0.02; Youden associated criteria ≤34.3 counts/µl. **(C)** Pearson correlation analysis was performed for PAds between BC200 serum levels and serum total calcium (p=0.03). The Pearson correlation coefficient, *r* value, is indicated in the graph. AUC, Area Under the ROC Curve; ROC, Receiver-Operating Characteristic.

We started this study analyzing BC200 expression in the serum of 27 PAds and 4 PCas by dPCR. BC200 counts were significantly higher in the serum of PCas compared to PAds, with a mean of 38.8 counts/µl versus 24.8 counts/µl, respectively (**Figure 1A**). Using the ROC analysis and the Youden’s statistic, we determined the cut-off able to discriminate PAds from PCas, which corresponded to 34.3 counts/µl (**Figure 1B**; p=0.02; Sensitivity=93%; Specificity=75%). According to this value, 24 out of 27 PAds and 3 out of 4 PCas were correctly classified (p=0.002; RR=6.8). Despite the statistical significance, we could observe that the sample with the highest level of BC200 was a PAd (PAd7; **Figure 1A**; blue square). We then looked at the clinical phenotype correlated of PAd7 and we noticed that it was characterized by the highest plasma PTH levels (**Table 1**).

Therefore, we correlated BC200 counts with PAds clinical and biochemical parameters. BC200 counts were positively correlated with serum total calcium (**Figure 1C**).

### Metastatic PCas Tissues Show Higher BC200 Levels Compared to Non-Metastatic Carcinomas

**Figure 2:**
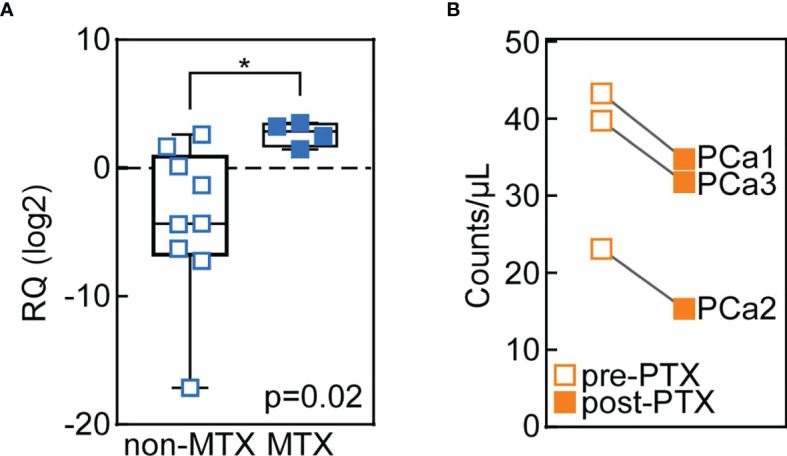
BC200 is overexpressed in tissues from metastatic carcinomas and its circulating levels decrease after parathyroidectomy (PTX). **(A)** BC200 levels were analyzed through qRT-PCR in parathyroid tissues from non-metastatic (MTX) PCas (n=9; blank squares) and MTX metastatic PCas (n=4; full squares). Whiskers represent min to max values. *p=0.02 from Mann-Whitney U-test. **(B)** Circulating BC200 levels are reduced after PTX (full squares) in PCa patients. BC200 counts/µl were measured using dPCR in the serum of 3 PCa patients, of which pre- and post-PTX samples were available.

Several *in vitro* evidence depicts BC200 as an oncogene involved in cancer cell migration, invasion and proliferation (21). To verify whether BC200 plays a role in cancer cell motility also *in vivo*, we analyzed BC200 expression in tissues from metastatic (n=9) and non-metastatic (n=4) parathyroid carcinomas. Confirming this hypothesis, we found that BC200 levels were upregulated in metastatic PCas compared with the non-metastatic ones (**Figure 2A**), supporting a role for BC200 in microenvironment invasion by parathyroid cancer cells.

### BC200 Serum Levels Are Decreased After Carcinoma’s Parathyroidectomy

Lastly, we analyzed circulating BC200 expression by dPCR in the pre- and post-operative serum samples from three PCa patients. The long non-coding RNA counts were significantly reduced after parathyroidectomy (PTX; **Figure 2B**).

This latter result further supports the use of BC200 as a non-invasive biomarker of PCa, useful for both initial disease diagnosis, staging and, possibly, clinical follow-up.

## Discussion

Here, we report novel observations about the lncRNA BC200 relevance in parathyroid tumors. Particularly, we found that pre-surgery circulating BC200 levels are higher in patients with parathyroid carcinomas than in those with benign adenomas. Importantly, BC200 expression in patients’ circulation drops decrease after the successful surgical removal of the cancer. Further, in PAd patients BC200 expression is directly correlated with increased serum calcium levels, a clinical parameter of active disease. Therefore, these results indicate that serological BC200 can be accurately measured in the circulation of patients with parathyroid tumors, and can be used to complement the clinical algorithm to correctly diagnose and follow subjects affected by parathyroid carcinoma.

Then, we tested the hypothesis of BC200 correlation with tumor aggressiveness by measuring its mRNA levels in non-metastatic and metastatic parathyroid carcinomas’ tissues. In line with its supposed oncogenic role, BC200 is more expressed in the lesions from metastatic PCas than in non-metastatic carcinomas, suggesting a possible involvement of BC200 in PCas’ metastatic process and invasiveness.

We previously reported that BC200, measured in parathyroid tissues, was able to discriminate benign from malignant parathyroid lesions. Now we extend this information providing novel evidence of the use of BC200 as a non-invasive biomarker of PCa. Despite the limited sample series, our results confirm the positive correlation of BC200 expression with disease severity. The use of a highly accurate methodology such as the digital PCR may rapidly translate, if confirmed in a larger cohort, these results into a point-of-care test to support the clinical and histological diagnosis of PCa versus PAd, and the follow-up of PCa patients. This could be crucial in the management of patients affected by parathyroid tumors, a complex class of diseases that lacks accurate biomarkers, especially of the non-invasive kind (24).

PCas are one of the rarest endocrine malignancies, representing <1% of parathyroid tumors (25). They are frequently characterized by markedly PTH hypersecretion and some patients experience hypercalcemia-related symptoms, with renal and bone involvement. The preoperatively diagnosis of this tumor is complicated and could be misleading since PCas often show clinical and molecular features shared by benign lesions and they are often indolent. The histological diagnosis of PCa is made on the presence of mitotic figures, capsular invasion, parenchyma gross infiltration, vascular invasion and, less frequently, distant metastasis (26). The complete resection of the tumor is the best treatment, even if it may not be curative. At the molecular level, mutations in few cell cycle-related genes such as CDC73, Rb and PRAD1, have been proposed as crucial in promoting parathyroid cells malignancy, but their role as PCas biomarkers is still uncertain. As a results, more than 50% of PCas recur at local or distant sites (27). Hence, there is an unmet need for a sensitive biomarker able to discriminate PCas from PAds and to stratify patients according to tumor aggressiveness and severity.

Epigenetic alterations represent a hallmark of cancer, mainly related to DNA methylation and histone post-translational modifications (28). These alterations, together with non-coding RNAs, make a great contribution to endocrine tumor development (29, 30). In parathyroid tumors, the hypermethylation of the tumor-suppressor HIC1, together with RASSF1A, CDKN2B and APC genes, were described in PCas and could be an early event in parathyroid tumor development (31, 32). Our previous study reported a lncRNA signature able to distinguish parathyroid carcinomas from adenomas and normal glands. Particularly, we found the lncRNA BC200 as the most upregulated one in PCas compared to other parathyroid tumour histotypes (11). Consistently with our findings, several works have reported a BC200 increase in many cancer types, where its overexpression promoted cell proliferation, metastasis formation and cancer aggressiveness (19, 33, 34). In addition, BC200 was detected in the peripheral blood of patients with invasive or metastatic breast cancer compared to disease-free patients (22).

Overall, our novel findings and previous data extend the knowledge on BC200 relevance in parathyroid tumors, supporting its role as a novel candidate biomarker in PCas diagnosis and follow-up.

## Data Availability Statement

The original contributions presented in the study are included in the article/supplementary material. Further inquiries can be directed to the corresponding authors.

## Ethics Statement

The studies involving human participants were reviewed and approved by Ethical Committee of Scientific Institute San Raffaele Hospital (#CE40/2019). The patients/participants provided their written informed consent to participate in this study.

## Author Contributions

AM and VV studied concept and design. AM and GP performed experiments. AM, SC, and VV analyzed and interpreted data. AM and GP acquired data and technical material. FC, SB, EP, VG, CV, GT, LV, CB, and SC provided tumor specimens and clinical information. AM and VV drafted the manuscript. SF and SC obtained funding. All authors critically revised and approved the final version of the manuscript.

## Funding

This work is supported by the Ricerca Corrente Program 2021 (from the Italian Ministry of Health) to SF and SC.

## Conflict of Interest

The authors declare that the research was conducted in the absence of any commercial or financial relationships that could be construed as a potential conflict of interest.

## Publisher’s Note

All claims expressed in this article are solely those of the authors and do not necessarily represent those of their affiliated organizations, or those of the publisher, the editors and the reviewers. Any product that may be evaluated in this article, or claim that may be made by its manufacturer, is not guaranteed or endorsed by the publisher.

## Abbreviations

250HD, serum 25-hydroxyvitamin D levels; AUC, Area Under the Curve; BCYRN1/BC200, Brain Cytoplasmic RNA 1; CCND1, Cyclin D1; CDC73, Cell Division Cycle 73; dPCR, digital PCR; lncRNA, long non-coding RNA; PAds, Parathyroid Adenomas; PCas, Parathyroid Carcinomas; PTH, Parathyroid Hormone; ROC, Receiver-Operating Characteristic; RR, Risk Ratio; WHO, World Health Organization.
